# A Red-Light Running Prevention System Based on Artificial Neural Network and Vehicle Trajectory Data

**DOI:** 10.1155/2014/892132

**Published:** 2014-11-04

**Authors:** Pengfei Li, Yan Li, Xiucheng Guo

**Affiliations:** ^1^Transportation School, Southeast University, 2 Sipailou, Nanjing 210096, China; ^2^School of Computing, Informatics and Decision Systems Engineering, Arizona State University, Tempe, AZ 85281, USA; ^3^Highway School, Chang'an University, The Middle Section of Southern Second Ring Road, Xi'an 710064, China

## Abstract

The high frequency of red-light running and complex driving behaviors at the yellow onset at intersections cannot be explained solely by the dilemma zone and vehicle kinematics. In this paper, the author presented a red-light running prevention system which was based on artificial neural networks (ANNs) to approximate the complex driver behaviors during yellow and all-red clearance and serve as the basis of an innovative red-light running prevention system. The artificial neural network and vehicle trajectory are applied to identify the potential red-light runners. The ANN training time was also acceptable and its predicting accurate rate was over 80%. Lastly, a prototype red-light running prevention system with the trained ANN model was described. This new system can be directly retrofitted into the existing traffic signal systems.

## 1. Introduction

Motorists face indecisiveness during the yellow and all-red clearance at signalized intersections. It is a composite result of the incompatible reactions to the changes of signal indicators and random safety perceptions among motorists. Such indecisiveness is a leading cause for signal violations at intersections. According to a research conducted by the University of California, Berkeley, 2.5 million accidents or 40% of all reported crashes in US were considered related to intersections in 2004 and 20% of these intersection accidents were signal-related [1], which can be interpreted a $13 billion loss annually [2]. In order to prevent the signal-related accidents, it is necessary to study the driver behaviors at intersections. Compared to the driver behaviors at other road segments (e.g., freeway or arterial links), the complexity of driver behaviors around intersections is that, during the yellow and all-red clearances, a driver will have to not only respond to signal changes but also interact with other adjacent vehicles to ensure safe maneuvers. As such, the driver behaviors at intersections can hardly be represented with analytical models.

The most severe problem caused by the signal violation is red-light running (RLR). The RLR is defined as a situation that approaching vehicles attempt to cross intersections during all-red or red phases. A RLR may be caused either by the driver's misperception of signal settings or simply by being distracted. Dilemma zone (DZ) is an area where the vehicles face indecisiveness of whether to stop or go at the yellow onset and it is commonly considered the primary reason for the RLR problem. Therefore, most of the RLR prevention systems in the past focus on modeling the driver behaviors in the DZ and countermeasures to protect the vehicles in DZ. Although success has been reported, some research also reported high RLR occurrences at congested and therefore low-speed intersections, where the DZ problem hardly exists [3, 4]. This finding implies that the RLR problem cannot be well addressed solely by mitigating the DZ issues. At congested intersections, the drivers may be distracted or just lose their respect to signal after excessive delays. They might determine to cross the intersection during the yellow, even though there is a RLR risk, so as to avoid further waiting. These intuitive explanations have little to do with the dilemma zone but significantly contribute to the RLR problem. After an extensive literature review, we concluded that there are no RLR prevention systems that could address the aforementioned situations because nearly all the existing RLR prevention systems, or collision avoidance systems, were based on vehicles' kinematics which did not take into account all possible reasons for the RLR issues. The RLR prevention system developed in this paper was based on the ANN technology. The ANN technology has been widely used to approximate complex system behaviors. In our system, variants of ANN networks were extensively trained to approximate the driver behaviors during yellow and all-red at intersections and the trained ANN model was used to predict if an approaching vehicle would become a red-light runner and take some preventive measures accordingly.

**Figure 1:**
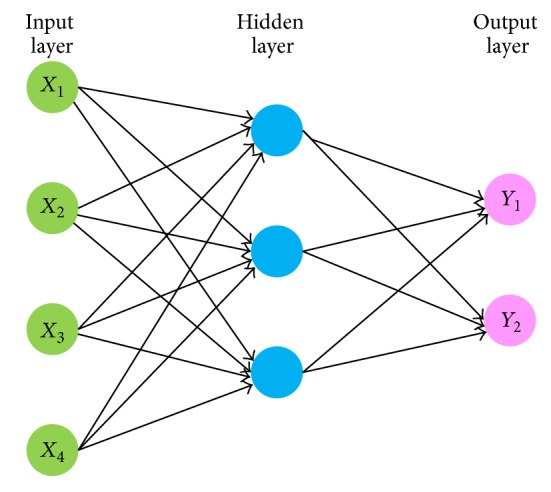
An illustration of classic three-layer neural network structure.

The development of ANN was initially inspired by understanding biological learning systems, such as human brains, but has been divided into two groups at present: one focuses on using ANNs to model biological process and the other focuses on developing effective machine learning algorithms [5]. The ANN is one of the most commonly used methods to approximate behaviors of complex systems. Typical ANNs are composed of a web of interconnected “neurons” (also called “nodes” or “processing units” in other literature) (see Figure 1). Mathematically, an ANN network is intended to approximate complex systems with weighted composition of relative simple functions which are further approximated with weighted composition of other simple functions and so on. A well-designed ANN should be able to contain sufficient system complexity.

In this paper, the authors first analyzed several ANN models to approximate the driver behaviors regarding the RLR problem and then illustrated the potential of using the ANN model to address the RLR problem. Second, a conceptual RLR prevention system was designed and evaluated. This paper is structured as follows: in the first section, the authors explained the structures of several popular ANN network variants, their advantages, and possible shortcomings; secondly, based on the vehicle trajectories data, the authors designed, trained, and then selected the most efficient ANN models as the fundamental predicting model to present the driver behaviors during the yellow and all-red clearance; this model served as the fundamental model in predicting possible RLR events. Lastly, the authors also discussed how to develop and retrofit this new system into the existing traffic signal systems.

## 2. Literature Review

The literature review was divided into two groups and reviewed, respectively: literature on the ANN and literature on the ANN's application to the traffic studies.

There is rich literature on the ANN theories and new research efforts are still being dedicated to this research today. Therefore this review can only cover a small portion of all related literature. As far as model specification is concerned, the ANNs are determined by three kinds of parameters:

- the interconnection pattern between different layers of neurons;
- the activation function that converts a neuron's weighted input to its output activation;
- the learning process for updating the weights of the interconnections.

### 2.1. Interconnection Patterns between Neurons

**Figure 2:**
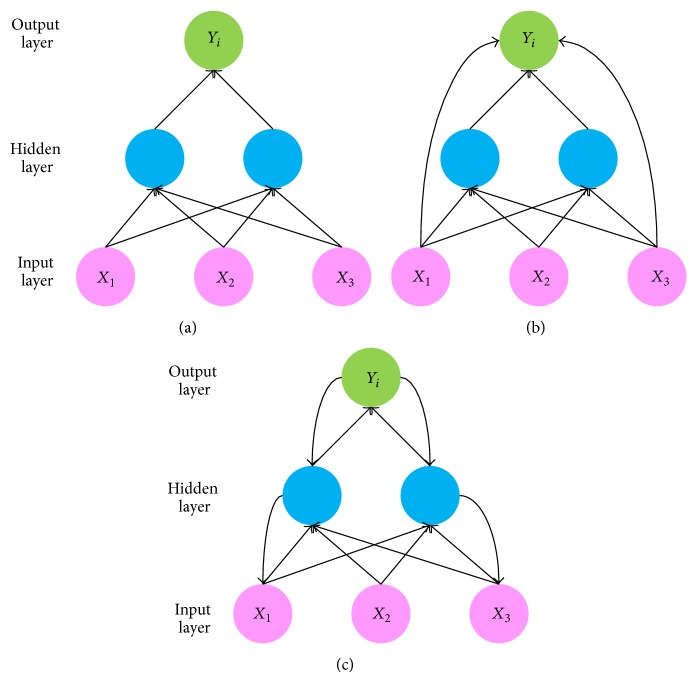
Three types of neural network connections.

According to the interconnection pattern between ANN neurons, the ANNs can be divided into feedforward and recurrent neural networks (RNNs). The connections between neurons in feedforward ANNs are acyclic like in Figure 2(a); a variant of feedforward ANN is the neural network with shortcuts in which some connections skip intermediate layer(s) like in Figure 2(b). In contrast, the connections in the RNNs can form circles and therefore use internal memory to process the inputs series as in Figure 2(c).

The* feedforward neural network* is relatively simple and commonly applied to various fields. McCulloch and Pitts are recognized as the founder of the ANN concept and designed the first neural network by combining many simple processing units together to increase in computational power [6]. The first influential feedforward neural network model can be traced back to the “perceptron” defined by Rosenblatt [7], which is a linear classifier by calculating a linear composition of the inputs with (1) and then outputting 1 if the calculation is greater than certain threshold, otherwise −1. Training a perception is a process of choosing values for the weights in the space *H* of all possible weight vectors:

(1) ox1,x2,…,xn =1if  w0+w1x1+w2x2+w3x4⋯+wnxn>0−1otherwise,

where *w*_*i*_ is the weight that determines the contribution of input *x*_*i*_.

From (1), the original perceptron is single-layer and can only express linear decision surface and the inputs have to be linearly separable. To overcome these shortcomings, the perceptron was extended to multiple layers, or the multilayer perceptron (MLP). The major difference between the original perceptron and MLP is that each neuron's output in MLP is a nonlinear and differentiable function (namely, activation function) of its inputs [8]. The MLP's nonlinear feature allows for representing more complex systems. Later, Werbos [9] and Rumelhart et al. [10] developed efficient backpropagation training algorithms for the MLP which significantly extend the MLP's applicability in various fields.

It is apparent that the feedforward neural network treats all the data as new and cannot discover the possible temporal dependence between samples. This shortcoming sometimes needs a feedforward neural network to be extended to a rather large scale to approximate complex systems. In other words, the feedforward neural network has a memoryless structure. By contract the RNN allows for the internal feedback and is more appropriate to solve certain types of dynamic problems. Jordan introduced the first RNN which feeds the outputs back to the input vector with time delay [11]. In other words, the RNN output at time *t* will be used as part of input information at *t* + 1. Mathematically, the outputs of a three-layer Jordan network with *m*, *q*, and *n* neurons on the input layer, hidden layer, and output layer, respectively, are as follows:

(2) ot+1,j=Fβj,0+∑h=1qβj,hGγh,0+xt′γh+ot′δh,j=1,2,…,n,

where *x*_*t*_′, *o*_*t*_′ are vectors of input and output at time *t*; *δ*_*h*_ is the vector of the connection weights between *h*th hidden neurons and input neurons which receive lagged outputs; *β*_*j*_ = (*β*_*j*,1_, *β*_*j*,2_,…,*β*_*j*,*q*_)′ is the vector of the connection weights between the *j*th output neuron and all *q* hidden neurons; *γ*_*h*_ = (*γ*_*h*__,1_, *γ*_*h*,2_,…,*γ*_*h*,*m*_)′ is the vector of the connection weights between the *h*th hidden neuron and all *m* input neurons; *F* and *G* are the activation functions on the output layer and hidden layer, respectively; and *β*_*j*,0_, *γ*_*h*,0_ are biased terms to add the flexibility of activation functions.

Similarly, Elman designed a RNN that the hidden neurons are connected to input neurons with time delay as in (3) [12]. Consider

(3) $$\begin{array}{c} o_{t+1,j}=F\left(\beta_{j,0}+\overset{q}{\underset{h=1}{\sum }}\beta_{j,h}a_{t,h}\right),\;j=1,2,\ldots ,n,\\ a_{t+1,h}=G\left(\gamma_{h,0}+x^{\prime }\gamma_{h}+a_{t}^{\prime }\delta_{h}\right),\;h=1,2,\ldots ,q, \end{array}$$

where *a*_*t*_ = (*a*_*t*,1_, *a*_*t*,2_,…,*a*_*t*,*q*_)′ is the vector of lagged hidden-neuron activations; *δ*_*h*_ is the connection weights between the *h*th hidden neuron and all the inputs which receive lagged hidden neuron activations.

Obviously, (3) and (4) can be extended to feed multiple previous step output vectors observations back to the input vectors.

### 2.2. Choice of Activation Functions

The activation functions in neurons are the building blocks of an ANN model. Similar to the neurons in a biology system, the activation function determines whether a neuron should be turned on or off according to the inputs. In a simple form, such on/off response can be represented with threshold functions, also known as a Heaviside function in the ANN literature as follows:

(4) $$\begin{array}{c} G\left(\gamma_{h,0}+x_{t}^{\prime }\gamma_{h}\right)=\left(\begin{array}{cc} 1, & \text{if}\;\;\gamma_{h,0}+x_{t}^{\prime }\gamma_{h}\geq 0\\ 0, & \text{if}\;\;\gamma_{h,0}+x_{t}^{\prime }\gamma_{h}<0 \end{array}\right), \end{array}$$

where *c* is the threshold and the remaining variables are defined previously. In some complex systems, the neurons may also need to be bounded real values. It is common to select sigmoid (S-shaped) and squashing (bounded) activation functions. It is also required that an activation function is bounded and differentiable. The most used two sigmoid functions in the ANN models are the logistic function and hyperbolic tangent (Tanh) function. Equations (5) and (6) are their mathematical expressions:

(5) $$\begin{array}{c} G\left(\gamma_{h,0}+x_{t}^{\prime }\gamma_{h}\right)=\frac{1}{1+e^{-(\gamma_{h,0}+x_{t}^{\prime }\gamma_{h})}}. \end{array}$$

(6) $$\begin{array}{c} G\left(\gamma_{h,0}+x_{t}^{\prime }\gamma_{h}\right)=\frac{e^{\left(\gamma_{h,0}+x_{t}^{\prime }\gamma_{h}\right)}-e^{-(\gamma_{h,0}+x_{t}^{\prime }\gamma_{h})}}{e^{\left(\gamma_{h,0}+x_{t}^{\prime }\gamma_{h}\right)}+e^{-(\gamma_{h,0}+x_{t}^{\prime }\gamma_{h})}}. \end{array}$$

### 2.3. Learning Process to Update the Weights of Interconnections

Training ANNs can be divided into supervised training and unsupervised training. The supervised learning needs pairs of training samples and each pair is composed of inputs and desired outputs (i.e., observations). The learning process is to adjust the interconnection weights to reduce the difference between the inferred outputs from the ANN model and the actual observations whereas the unsupervised learning is to find hidden structure in unlabeled data with, for example, statistical inference. In the context of this paper, the authors only review part of influential supervised learning algorithms.

To effectively approximate the complex systems, the interconnection weights in the ANNs have to be estimated with the existing observations. A simple example with only one single target output *y* and the network function *y* = *f*_*G*,*q*_(*x*; *θ*) is used to illustrate how to update the weights. *θ* is the vector of interconnection weights.

After the activation *G* and the structure of hidden layers are determined and a training sample of *T* observations is given, the optimal *θ* can be obtained by minimizing the mean squared error (MSE) in (7), which can be obtained with the first order differentiation of (7) (i.e., (8) and (9)):

(7) $$\begin{array}{c} \frac{1}{T}\overset{T}{\underset{i=1}{\sum }}{\left(y-f_{G,q}\left(x;\theta \right)\right)}^{2}, \end{array}$$

(8) $$\begin{array}{c} E\left[\nabla f_{G,q}\left(x;\theta \right)\left(y-f_{G,q}\left(x;\theta \right)\right)\right]=0, \end{array}$$

where *f*_*G*,*q*_(*x*; *θ*) is the gradient vector of *f*_*G*,*q*_ with respect to *θ*. Rumelhart et al. designed a recursive gradient-descent-based algorithm to estimate the $\hat{\theta }$ as follows [10]:

(9) $$\begin{array}{c} {\hat{\theta }}_{t+1}={\hat{\theta }}_{t}+\eta_{t}\nabla f_{G,q}\left(x;{\hat{\theta }}_{t}\right)\left[y_{t}-f_{G,q}\left(x;{\hat{\theta }}_{t}\right)\right], \end{array}$$

where *η*_*t*_ is the learning rate and (9) is so called backpropagation algorithm and is a generalized form of the “delta rule” of single-layer perceptron model [5]. Some famous variants of the backpropagation algorithm include the* Quickprop* algorithm [13] and the Resilient Backpropagation (*RPROP*), an adaptive learning algorithm [14].

### 2.4. Complexity Regularization

Given a fixed training sample, an ANN with excessive hidden neutrons will overfit the data whereas the ANN with insufficient neutrons cannot capture all of systems' properties and become unstable. In analogy to the linear programming problem, the excessive neurons issue is like more equations than the variables whereas the insufficient neurons issue is like more variables than equations.

One idea is to begin an ANN with zero hidden neurons instead of fixing the ANN structure at first and then insert hidden neurons as needed until the MSE can be reduced to an acceptable level. One of commonly accepted such algorithms is the cascade-correlation (CC) developed by Fahlman and Lebiere [15]. The initial CC neuron network contains zero hidden neuron and therefore it is likely that the target MSE cannot be reached even with a large size of training data. Secondly, several so-called candidate neurons are created and they are only connected to all input neurons and existing hidden neurons with random weights; the third step is to train the weights of neuron candidates to maximize the correlation between the candidate hidden neurons' activations and overall network errors, which is calculated with (10). Thirdly select the candidate neuron with the highest correlation, freeze its connection weights (i.e., unchangeable during the later training process) to the input neurons, and connect it to the output neurons with random connect weights. At this point, the original CC network grows by one more neuron and lastly the new CC network is trained again to minimize the MSE. If the target MSE is reached, then the training process ends; otherwise, go back to step 2 and repeat until the target MSE is reached. Obviously, the final CC network contains multiple single-neuron hidden layers:

(10) $$\begin{array}{c} C=\frac{\sum_{o}\left|\sum_{p}\left(h_{p}-\langle h\rangle \right)\left(e_{op}-\langle e_{o}\rangle \right)\right|}{\sum_{o}\sum_{p}{\left(e_{op}-\langle e_{o}\rangle \right)}^{2}}, \end{array}$$

where *h* is the hidden neuron activation; *e* is the network error; 〈*h*〉, 〈*e*_0_〉 are means.

As for the ANN's applications to the traffic studies, it is still in its infantry. Lu et al. developed a neural network based tool to filter and mining the highly skewed traffic data [16]; Huang utilized the wavelet neural network to forecast the traffic flow and the results reveal the forecasting accuracy was improved compared to the traditional methods [17]; Chong et al. deployed the feedforward neural network to train the driver in simulation based on the naturalistic data and the results showed that the driving behavior is closer to the actual observation than the traditional car-following models [18]. Jia et al. trained an ANN-based car-following model with the data collected via a five-wheel system. The inputs include speed of following vehicle, relative speed, relative distance, and desired speed. The output vector includes the acceleration of the following vehicle [19]. Panwai and Dia developed a similar backpropagation-neural-network-based car-following model in AIMSUN, commercial microscopic software. Compared with the embedded car-following model in AIMSUN, the new car-following model is 20% better in terms of errors reduction [20].

## 3. Significance of the Research

Many severe crashes occur at signalized intersections today due to signal violations and so it is important to study the red-light running prevention at intersections. Compared with the other traffic segments, the driver behaviors close to signalized intersections are more difficult to represent by models due to the random individual vehicle's decision, intensive interactions between vehicles, and the complex feedback mechanism between vehicles and the traffic signal system. The driver behaviors study at signalized intersections will help identify the possible reasons of certain unsafe vehicle maneuvers, such as the RLR, and eventually help develop countermeasures to mitigate the safety hazards.

In the past, it is commonly assumed that the RLR is caused by the dilemma zone and therefore most of the related research of the red-light running issue focused on how to minimize the vehicles in the dilemma zone. However, some recent research on vehicle trajectories during yellow and all-red clearance reveals that vehicles may still run red lights at low speeds in which the dilemma zone issue hardly exists. This finding implies that there must be other factors than the dilemma zone to contribute to the red-light running. Intuitively, drivers at congested intersections may be more likely to take the RLR risk to cross the intersection in order to avoid further waiting. Or the drivers may be just distracted and fail to observe the traffic lights. Obviously, the reasons for RLR are complex and difficult to be represented with the traditional modeling method. Meanwhile, although it is difficult to precisely analyze the reasons for the individual RLRs, the red-light runners may still share some common kinematic patterns, such as shorter headways from their leading vehicles or faster speeds at yellow onsets. These kinematic features can be retrieved from the vehicle trajectory data collected via the radar, video imaging detectors, or the connected vehicle technology in the future.

In this paper, the authors explored various ANN networks to approximate the driver behaviors during the yellow and all-red clearances. The inputs of ANNs (i.e., vehicles' kinematic features) were captured and calculated based on the vehicles' trajectories during the yellow and all-red clearance. The well trained ANN model then served as the fundamental predictive model to identify the possible red-light runners. The collision avoidance measures were activated then to avoid potential crashes.

## 4. Methodology

### 4.1. Problem Representation

#### 4.1.1. ANN Model Inputs

It is assumed that a potential RLR event begins at the yellow onset when a driver has to decide whether to cross or stop according to his safety perception. The safety perception is psychological and determined by many factors. However, only the four most important factors were taken into account as the ANN input candidates: distance-to-intersection (DTI), travel speed (*v*_*i*_), the number of front vehicles before the stop line, and headway from the leading vehicle (*h*_*i*_). These parameters were reportedly effective to represent a driver's status at the yellow onset in other literature [21]. No lane changing was considered in the fact that it was rare according to our field observations.

#### 4.1.2. ANN Model Outputs

In general, there are two possible time moments able to be used to tell an occurrence of RLR: the all-red onset and at the all-red end. If the all-red onset is used, a subject vehicle would be considered a red-light runner when it has not reached the stop line but cannot completely stop according to its distance to the stop line, speed, and maximal possible deceleration. If the all-red end is used, a subject vehicle is considered a red-light runner when it is still within the intersection when the all-red clearance expires. In the all-red-end-based method, two factors are considered relevant regarding the ANN outputs, the DTI and speed.

Using the all-red onset would have to assume that driver becomes the red-light runner only when it cannot stop. However, this assumption is questionable because a slow driver may still want to take the RLR risk to cross the intersection or it may just be distracted and become a red-light runner. Therefore, in this paper, a vehicle's status at the all-red clearance end was used to measure the RLR event. Two types of outputs were used: (1) classifier: a vehicle was labeled as a run-light runner if it was still within the intersection at the all-red clearance end, regardless where it is exactly; (2) the vehicle's location and speed were observed within the intersection at the all-red end. These two output variants were evaluated, respectively, and compared later.

### 4.2. ANN Model Design

#### 4.2.1. ANN Structure

Since the driver behaviors during the yellow and all-red are independent from cycle to cycle (i.e., the drivers are not aware of other vehicles' maneuvers in previous cycles), the feedforward neuron network was selected due to its memoryless property. In terms of the number of hidden neurons, two ANN structures were used and compared: standard feedforward neuron network with multiple hidden layers and the cascade-correlation (CC) neuron network which iteratively inserts new hidden neurons until the CC can achieve the desired MSE.

#### 4.2.2. Training Algorithms

A variant of the generic backpropagation algorithm is used to train the ANNs, namely,* QuickProp*. The readers can refer to the literature [13] for more details. The weights were updated once with (9) after all the sample pairs were fed in. In other words, the standard backpropagation algorithm in this paper was offline and the weights were updated only once after each epoch.

### 4.3. Experiments Design

Although the RLR event is a severe safety problem, its occurrence is relatively rare at most intersections in reality. In order to capture real RLR samples, it would take several months to collect data and the data processing would be tedious too. As such, we determined using microscopic simulation to create RLR samples. The challenge of using simulation to train the ANN network was how to adjust the simulation settings so that the real driver behaviors could be accurately reflected in simulation. In this paper, we used PTV VISSIM simulation engine and carefully calibrated the VISSIM's car-following model and stop-or-go responses to signal changes with the vehicle trajectory data at the Peppers Ferry intersection in Christiansburg, Virginia, which was collected with a high performance data acquisition system. The data were choreographed and recorded by a customized hardware package. The data included synchronized vehicle trajectories, signal phases states, and error messages and were stored at 20 HZ to a binary file. The sensing system was composed of radar, signal sniffer, and video imaging systems.  Table 1 illustrates vehicle's trajectory data snapshots at the yellow onset and after all-red clearance.

Each vehicle's trajectory and its stop-or-go decision at the yellow onset were summarized. Then vehicles' speed distribution, average headway, still headway, acceleration distribution, and other information were summarized [22]. Then they all were input into the simulation settings. After these adjustments, vehicles' behaviors in simulation were very close to the field observation. However, it was found that few RLR events occurred in simulation and therefore we further reduced the drivers' attention to their front vehicles and to traffic signals. This change generated more red-light runners in simulation and significantly increased RLR samples for the following ANN training.

In reality, either current vehicle trajectory detectors or future connected vehicle technology has a discovery range from 200 meters to 400 meters [23]. Therefore only those vehicles whose distance to the stop line was less than 100 meters were monitored and the status (i.e., DTI_*i*_, *v*_*i*_,* and h* ^*i*^) of each monitored vehicle was archived at the all-red end if it was within the range during the yellow and all red. The 100-meter area can be translated into 5.5 seconds to 6 seconds to stop line where drivers' indecisiveness begins at the yellow onset.

The speed limit and traffic volume were 60 km per hour and average 1500 vph on the link and two simulation runs were conducted and lasted until 300 RLR events were captured.

Faster training can be achieved by normalizing the inputs and outputs. The captured vehicles' DTI was normalized by DTI_*N*_ = DTI/*L*_*d*_ where the *L*_*d*_ is the length of discovering area and 100 meters in this paper. Similarly, the speed, headway, and the number of front vehicles were also be normalized in a form of *a*_*N*_ = (*a* − *a*_min⁡_)/(*a*_max⁡_ − *a*_min⁡_) where the *a*, *a*_min⁡_, and *a*_max⁡_ are the original value, the observed maximal value, and observed minimal value, respectively. The data from one simulation run were used to train the ANNs and the data from the other independent simulation run were to validate the training effects and prevent the overfitting issue.

## 5. ANN Training and Results Evaluation

Multiple experiments were conducted and the results were compared to determine the best ANN model to predict the individual vehicle's RLR possibilities. The ANN training process is usually long but once the training is finished, the well trained ANN model is essentially an analytical model and so it is fast enough for all kinds of online applications.

### 5.1. Scenario One: Input Data Are Combined with Red-Light Runners and Regular Vehicles

*Step 1*. Train and compare various ANNs with different compositions of input variables, output variables, and network structures. The training algorithm was the standard backpropagation algorithm as in (9) with the learning rate 0.7 and the stopping MSE was 0.005. The activation functions were set as the Tanh functions (6) for both hidden neurons and output neurons. Preliminarily, sixteen options were generated with various compositions of inputs and outputs. The underlying rationale was that some input variables may contribute more to the RLR problem than the others and it is needed to only capture the most important factors to avoid overcomplicating the problem. In addition, the output variants are useful for various collision avoidance strategies.

Given that we had little prior knowledge about how many hidden layers and neurons of the MLP network were sufficient to approximate the RLR problem, it was wise to start with the cascade-correlation (CC) network which gradually adds hidden neurons while learning and the final CC structure can help us to better understand the ANN's necessary complexity.  Table 2 describes the configurations of all the sixteen options. After some preliminary tests, the maximum of hidden neurons in the CC model was set as 100 because more neurons made the training excessively long with only limited further MSE reduction. The MLP structure was designed as three hidden layers and each hidden layer contains 10 hidden neurons.

**Figure 3:**
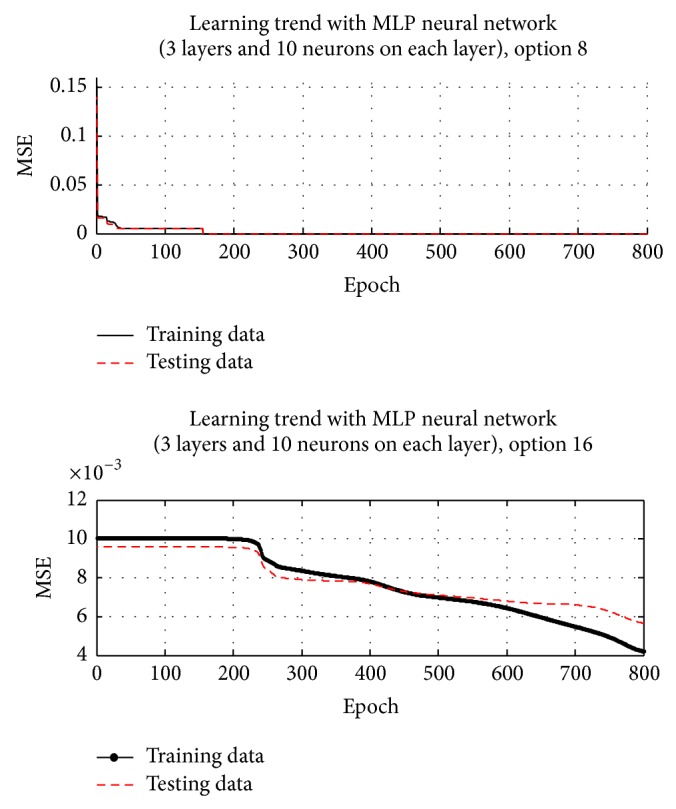
Training trend under the Option 8 and Option 16 models.

Table 3 is the ranking in the minimum MSE (i.e., the effectiveness of approximation). From Table 3, only Options 8 and 16 could reach the target MSE (0.005) and therefore be selected as the candidate model and then go to the next step: model validation. The remaining options stagnated before reaching the desired 0.005.  Figure 3 reveals the learning trends of Option 8 and Option 16. Option 8 and Option 16 had no overfitting issues before reaching the target MSE since the test MSEs kept decreasing in the training process.

*Step 2 (model validation with a new set of data). *After the models were trained and two candidate models were selected, these two ANN models were coded into the RLR prevention system to serve as the fundamental predictive model. In the next section, the development of the RLR prevention system is described in detail. Before the system was deployed in the real world, the ANN model was first tested to justify its accuracy in predicting red light runners according to their kinematic patterns at the yellow onset. The RLR prevention will not work unless the red-light runner prediction is accurate enough. Two types of errors were evaluated: Type I error: a regular vehicle was reported as a red light runner; Type II error: a red-light runner fails to be predicted. The new set of data contains 1500 samples which includes 1450 regular vehicles and 150 red-light runners.  Table 4 reveals the results of Type I and Type II errors.

From Table 4, it seems that both ANN models had low rates of false alarms (i.e., Type I error) but were not effective in predicting the red-light runners (i.e., high rates of Type II error). It makes sense because the vast majority of data was composed of regular vehicles and therefore the ANN models overwhelmingly learn the patterns of regular vehicles compared to the red-light runners. Therefore if the mixed data (regular and red-light runner) were used for training, the false alarm rate was low whereas the accurate rate of predicting RLR events was low due to lack of enough samples. In order to improve the RLR predicting effectiveness, Scenario Two was designed which only contains the red-light runner data.

### 5.2. Scenario Two: Input Data Only Contains the Red-Light Runners

**Figure 4:**
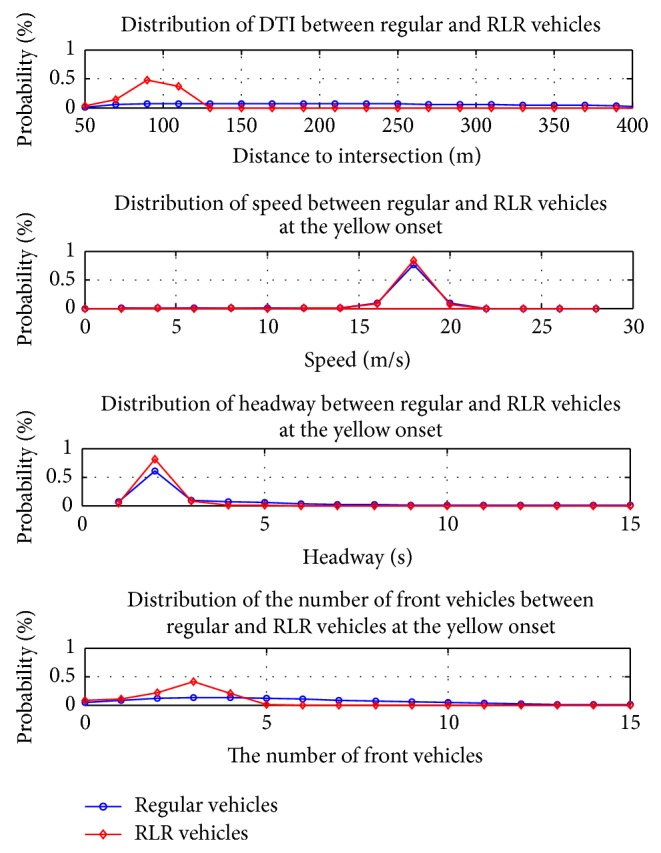
Comparison between regular vehicles and RLR vehicles.

Similarly, Scenario Two was also divided into two steps. The Options 9~16 in Table 2 were no longer suitable since all the data were for red-light runners. From the previous experience in Scenario One, all four relevant inputs were selected and the vehicle's location at the all-red end was selected as the ANN output. We compared the four relevant four inputs of regular vehicles and red-light runners and displayed the results in Figure 4. It seems that most red-light runners were 50 meters to 130 meters away from intersection at the yellow onset which means 3 to 6 seconds to the intersection. It was also found that the RLR vehicles tended to have slightly higher speeds, shorter headways, and fewer front vehicles at the yellow onset. These phenomena make sense because the RLR vehicles are intuitively more aggressive than regular vehicles and these findings also supported our fundamental speculations that the RLR vehicles could be distinguished from regular vehicles according to their features at the yellow onset.

Learning from Scenario One, we found that the number of neurons should be at least 100 in order to capture the key patterns of red-light runners and reduce the MSE to the desired level. After several preliminary experiments, fully connected and with five hidden layers with 20 neurons on each hidden layer. The activation functions of all neurons were the symmetric sigmoid as in (6).

**Figure 5:**
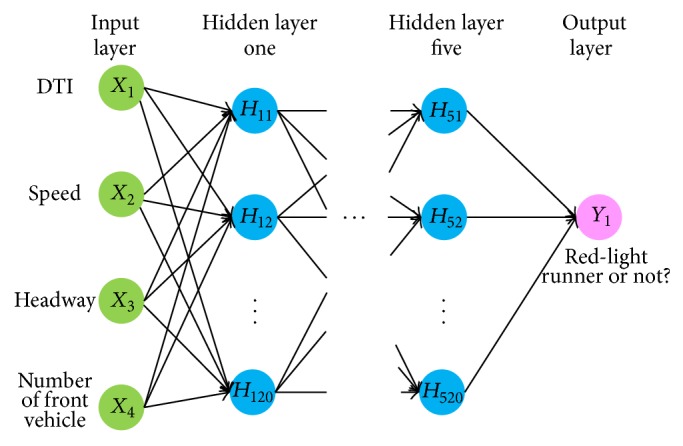
Structure of ANN network for training.

**Figure 6:**
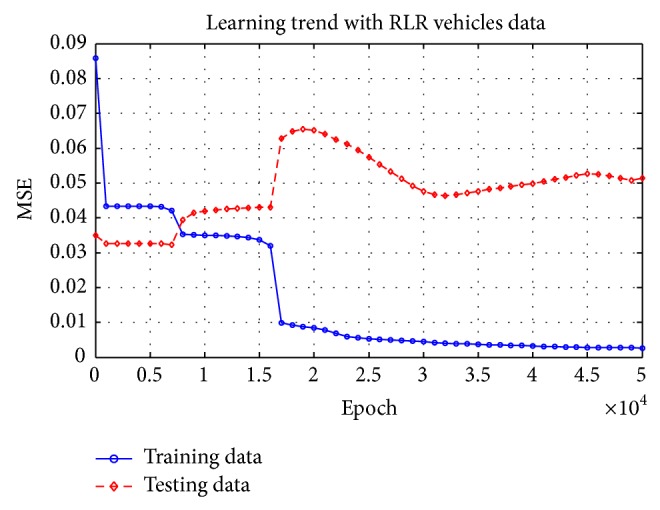
Training trend with the red-light running data.

*Step 1 (collected 300 RLR event samples).* Consider that the sample size was smaller and therefore the target MSE was reduced to 0.  Figure 6 reveals that model in Figure 5 converged quickly and then became stagnated while the test MSE begins to increase. Therefore the final ANN model was selected at the 7000th epoch.

*Step 2 (model validation with a new set of mixed data containing 300 new RLR events and 7,000 regular vehicles).* Table 5 shows the predicting accuracies of the trained ANN models. Compared to Table 4, the new ANN model could significantly reduce Type I and Type II errors in the RLR prediction. This makes sense because the ANN was trained with RLR samples only and therefore the accuracy of predicting RLR events would clearly increase. Meanwhile, since this is a binary identification problem, the regular vehicles' identification accuracy will also be increased accordingly. The total training time was about two and half hours with a standard desktop PC, which was acceptable.

**Figure 7:**
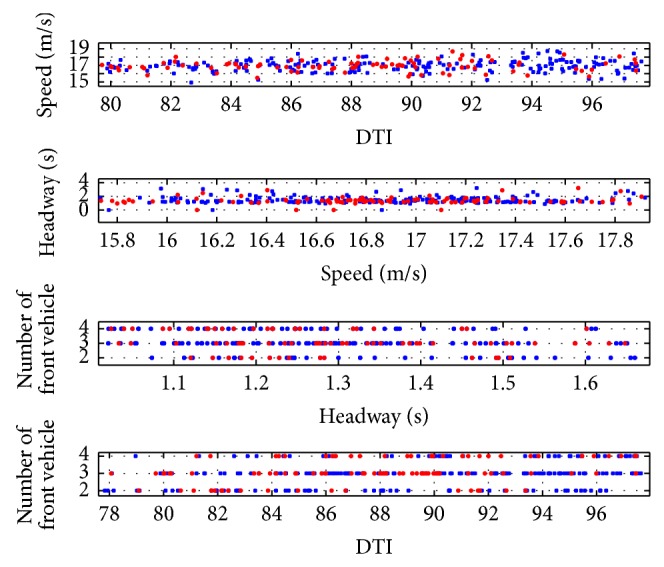
Plot of identified and unidentified RLR vehicles.

With all RLR samples, we further plot identified (blue in Figure 7) and unidentified samples (red in Figure 7), respectively, to seek the dominant factors in identifying the RLR vehicles. However, from Figure 7, none of the four factors were statistically effective to separate identified and unidentified groups. Therefore, the ANN model should not be further simplified such as excluding some selecting inputs. Otherwise the predicting accuracy of RLR vehicles would deteriorate.

## 6. Red-Light Running Prevention System

**Figure 8:**
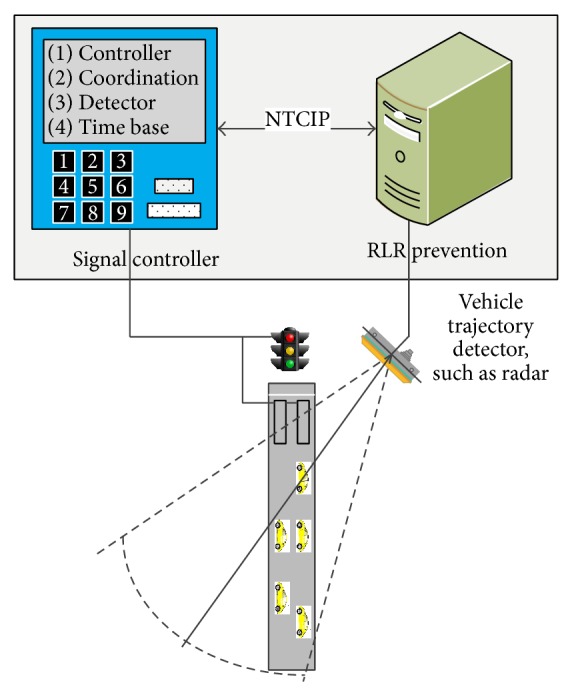
Architecture of the new RLR prevention system.

The challenge of developing such a system is that the ANN network will not be supported by any commercial signal control equipment at this stage and therefore some interfacing equipment must be designed to retrofit this new system into the existing traffic signal systems. Nowadays, most traffic signal controllers in the field are compliant with the National Transportation Communication for ITS protocols (NTCIP) [24]. Through the serial port or Ethernet port on a signal controller, it is possible to override the current timings to prevent the possible RLR-related collisions, such as extending the all-red clearance or extending the current green. As in Figure 8, after the ANN model is trained, the ANN model will be ported to a hardened computer and become a module of the RLR prevention system. The hardened computer will also be connected to a vehicle trajectory detector located at the far end of intersections via the standard Ethernet, such as trajectory radar [25]. The radar will keep monitoring approaching vehicles and record their speeds, accelerations, and distance. At the same time, the hardened computer also keeps retrieving the signal phasing message from signal controller via the NTCIP 1202 object, such as “OID: 1.3.6.1.4.1.1206.4.2.1.1.4.1.4.1” (Phase Group One Green Status) to understand the phasing status. When a green phase is about to end, the RLR prevention system will examine the approaching vehicles' speeds, distances to stop line, headway, and other kinematic parameters and then predict if the number of potential red-light runners is beyond a threshold with the ANN model. If so, the computer will send a “hold” NTCIP message to the signal controller, “OID: 1.3.6.1.4.1.1206.4.2.1.1.5.1.4.1” (Phase Hold) to override the current timing and extend the current green for another several seconds. After each extension, there is a minimum time interval for another green extension. At the same time, if the computer finds there are vehicles still within the intersection or some aggressive vehicles are impossible to completely stop after the all-red clearance, the computer will issue another “Phase Hold” command to prevent vehicles on other approaches from entering the intersection. Through these two types of safety countermeasures, the RLR event can be substantially reduced. In practice, the radar detector may lose tracking vehicles when they are totally stopped. However, the latest radio detection product can identify and estimate the vehicles' trajectories with satisfaction.

The ANN model should be retrained periodically, such as every three months, after sufficient new RLR samples are collected in the field. This way will ensure the system's effectiveness to the continuingly evolving traffic patterns.

## 7. Conclusions and Future Work

The red-light running is a leading cause for severe crashes at intersections and it has been assumed that the dilemma zone is the major reason for the RLR occurrence. However, recent research has revealed that the RLR occurrence is caused by not solely the dilemma zone but also many other factors. The complexity of modeling the RLR process is beyond most of the close-form analytical models. In this paper, the authors present the potential of the artificial neural networks to approximate the RLR process and predict the RLR occurrence according to vehicles' four statuses (DTI, speed, headway, and the number of front vehicles) at the yellow onset. This information can be obtained from the vehicle trajectory sensors or the connected vehicle technology in the future. From the multiple experiments, we concluded that using the data at the yellow onset as the input and the data at the all-red end as the output is the most effective while training the ANN networks. Using the well trained ANN model, we developed a prototype of RLR prevention system which can predict the potential red-light runners and take countermeasures accordingly.

The predicting accuracy is critical to the success of RLR prevention. With the properly selected inputs and outputs, the final ANN model can predict the RLR events with over 80% accuracy. Besides the two aforementioned countermeasures, other countermeasures are also possible, such as activating warning beacons to remind the potential red-light runners if necessary. Or, when the connected vehicle technology has a high market penetration in the future, the signal control system can send out customized warning messages to individual on-board units according to their specific status.

Although this new system has been proven effective in simulation, it must be further evaluated in the field before being fully deployed. Therefore we plan to deploy this prototype system in the field, fine-tune the ANN networks, and justify the systems' performance with the real RLR samples. Nevertheless, since the ANN model established in this paper does not depend on the vehicle kinematics or any traffic flow theories and the ANN model just retrieves the certain empirical features from the observed driver behaviors, it is expected that the performance of RLR prediction will not be significantly different between the simulation results and the filed observation data. In the future, we will plan to conduct more investigation on applying the artificial neural networks and other artificial intelligence technologies to the traffic safety improvement at signalized intersection.

## Figures and Tables

**Table 1:** Illustration of vehicle trajectory data snapshots.

Time	Phase	Veh_ID	Link	DTI	Speed	Headway	Front_veh
17:00:05	1_Y_start	8	1	97	17.1	2.6	2
17:00:11	1_AR_end	8	1	9	7.1	0.0	0

**Table 2:** Configurations of preliminary twelve ANNs.

	Input: yellow onset				Output: all-red end			ANN structure	
	DTI	Speed	Headway	Number of front veh.	DTI	Speed	Classifier	Fixed-structure	Dynamic cascade-correlation
Option 1	X	X			X	X		X	
Option 2	X	X			X	X			X
Option 3	X	X	X		X	X		X	
Option 4	X	X	X		X	X			X
Option 5	X	X		X	X	X		X	
Option 6	X	X		X	X	X			X
Option 7	X	X	X	X	X	X		X	
Option 8	X	X	X	X	X	X			X
Option 9	X	X					X	X	
Option 10	X	X					X		X
Option 11	X	X	X				X	X	
Option 12	X	X	X				X		X
Option 13	X	X		X			X	X	
Option 14	X	X		X			X		X
Option 15	X	X	X	X			X	X	
Option 16	X	X	X	X			X		X

**Table 3:** MSE ranking among various options.

Option	8	16	2	14	6	4	12	10	15	9	1	5	7	13	3	11
Min MSE	0.005	0.005	0.0058	0.0067	0.0087	0.0089	0.0089	0.0095	0.0098	0.0225	0.0243	0.0252	0.026	0.0268	0.0274	0.0274

**Table 4:** Results of data validation in scenario one.

		Observed	
		Positive	Negative
Calculated (Option 2)	Positive	11%	3%
	Negative	89%	97%

Calculated (Option 16)	Positive	11%	3%
	Negative	89%	97%

**Table 5:** Results of data validation in scenario two.

		Observed	
		Positive	Negative
Calculated	Positive	82%	18%
	Negative	17%	93%
